# Incorporating Non-Linear Epoxy Resin Development in Infusion Simulations: A Dual-Exponent Viscosity Model Approach

**DOI:** 10.3390/polym17050657

**Published:** 2025-02-28

**Authors:** Mohammad W. Tahir, Umar Khan, Jan-Peter Schümann

**Affiliations:** 1 Department of Computer Science, Edge Hill University, Ormskirk L39 4QP, UK; khanu@edgehill.ac.uk; 2 Westlake Esslingen GmbH, 73730 Esslingen am Neckar, Germany; ftjp@gmx.de

**Keywords:** glass fibres, liquid composite moulding, viscosity, cure behaviour

## Abstract

In the field of liquid composite moulding (LCM) simulations, a long-standing assumption has dominated–the belief in constant resin viscosity. While effective in many cases, this assumption may not hold for the infusion process, which lasts for an extended period. This impacts the mechanical properties of the cured epoxy, which are crucial for load transfer in polymer structures. The majority of epoxy resins operate on a bipartite foundation, wherein their viscosity undergoes dynamic alterations during the process of cross-linking. Temperature and cross-linking intricately interact, with elevated temperatures initially reducing viscosity due to kinetic energy but later increasing it as cross-linking accelerates. This interplay significantly influences the efficiency of the infusion process, especially in large and intricate moulds. This article explores the significant temperature dependence of epoxy resin viscosity, proposing an accurate model rooted in its non-linear evolution. This model aligns with empirical evidence, offering insights into determining the optimal starting temperature for efficient mould filling. This study presents an advanced infusion model that extends existing non-linear dual-split viscosity approaches by incorporating the experimental validation of viscosity variations. Unlike previous models that primarily focus on theoretical or numerical frameworks, this work integrates experimental insights to optimize infusion temperature for efficient resin infusion in large and complex parts. Building on these findings, a novel mould-filling technique is proposed to enhance efficiency and reduce material waste.

## 1. Introduction

**Figure 1:**
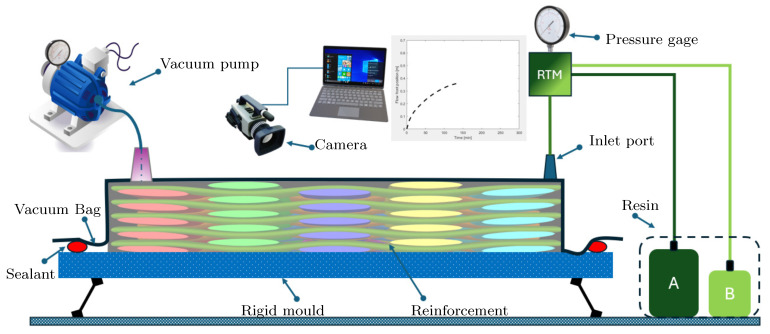
Schematic of the LCM. Different component of the resin to be mixed in specific proportion are shown with different colours.

Liquid Composite Moulding (LCM) stands out as a pivotal process in the fabrication of Fiber-Reinforced Polymer (FRP) structures. Despite the existence of alternative manufacturing methods [1,2,3], the traditional closed mould LCM process remains highly favoured in sophisticated applications, such as the production of maritime wind turbine blades, boats, aerospace components, etc. A schematic of a typical LCM process is shown in Figure 1. The ultimate properties of an FRP composite structure in the closed mould filling process are intricately linked to factors, including mould filling conditions, comprehensive wetting of fibres, and well-regulated curing of the resin [4].

In the context of closed mould filling, challenges escalate, especially for extensive and complex moulds, where resin accumulation in certain regions becomes a pertinent issue. The exothermic nature of resin curing intensifies this concern, as accumulated resin generates additional heat, potentially leading to localised overheating of the mould, hence causing damage. Moreover, this phenomenon can compromise the structural properties of the FRP structure.

The Resin Transfer Moulding (RTM) process, commonly used for fabricating smaller components with shorter filling durations, ensures sufficient time for resin impregnation of reinforcement fibres before polymerisation or consolidation-impeding advancement occurs, and in small moulds, resin viscosity can reasonably be assumed to be constant for numerical modelling [5], as the mould-filling time supports this approximation. However in other methods, such as Resin Infusion under Flexible Tooling (RIFT), used for the production of gigantic parts [5,6], such an assumption can introduce substantial errors into the numerical solution of the problem.

**Figure 2:**
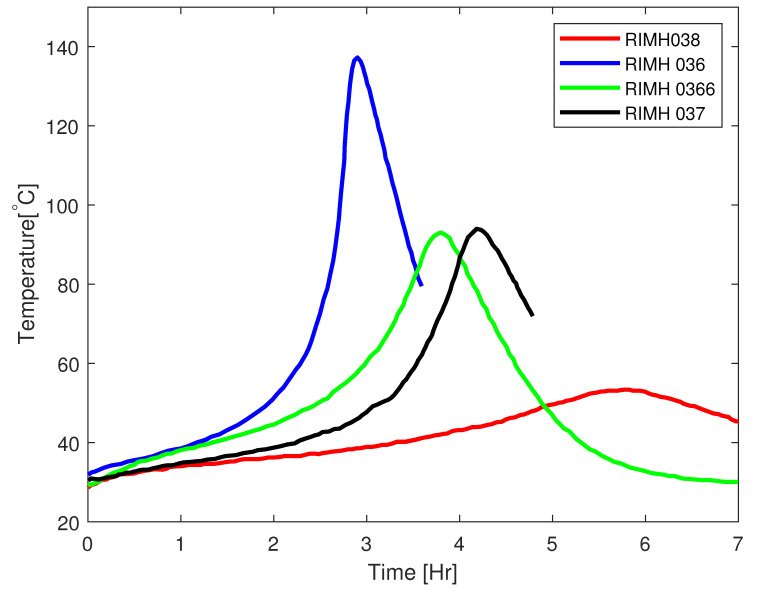
Plot of temperature development of RIMR 035c epoxy resin with four different hardeners redrawn from manufacturer datasheet.

In most advanced applications, epoxy resin is used as a matrix resin due to its superior mechanical properties [7]. For most epoxies, cross-linking begins as soon as they are mixed with the hardener, containing the cross-linking sites. The rate of cross-linking, however, is sufficiently low for a larger time window for many small-to-mid size applications, as shown in Figure 2.

Despite the existence of certain epoxy formulations exhibiting diminished reactivity and, consequently, slower viscosity evolution, as illustrated in Figure 2, polymerisation may still progress to a stage where the resin fails to reach critical regions within the mould or ceases its movement entirely before complete mould filling occurs. This can result in either an incompletely filled mould or the presence of dry spots in the final structure. Dry spots can extend maintenance time, leading to material wastage and reduced mechanical properties of the structure. Thus, precise knowledge of the flow front and accurate mould fill time are crucial for process optimisation, product quality, and minimising environmental impact.

An additional pivotal determinant influencing the mechanical characteristics of a composite structure is the temperature history and processing conditions encountered during the epoxy resin infusion process [7,8]. Given the reduction in resin viscosity with rising temperatures—at least at the start of mixing ingredients, conducting the infusion at elevated temperatures appears advantageous. Nevertheless, the augmented temperature accelerates the cross-linking reaction, and the heightened cross-linking rate results in a further temperature increase due to the exothermic nature of the cross-linking process. Consequently, the resin’s viscosity undergoes a rapid escalation. Hence, it is imperative to identify an optimal starting temperature for the process to ensure the comprehensive filling of large moulds.

In a recent study, a phase field-based modelling approach was proposed to improve resin flow prediction, defect identification, and process optimisation [9]. Simacek et al. present a coupled process modelling approach for flow and transport phenomena in the LCM processing. Their methodology enhances resin infusion simulations by integrating volatile tracking and transport mechanisms. Gayot et al. develop a thermochemical model to predict temperature distribution in methyl methacrylate-based composite infusion, using the quasi-steady-state approximation for efficient computation and optimising mould heating to minimise void formation during polymerisation [10]. In another study, Huang. R modelled multiphase resin and air flow in a vacuum-assisted infusion, incorporating temperature changes while assuming constant viscosity, offering valuable insights for the infusion process [11]. Along with these studies, numerous models have been posited to elucidate the temporal evolution of viscosity in epoxy resins [12,13,14,15,16,17]. Nevertheless, these investigations have hitherto overlooked the specific impact of viscosity development during the implementation of RTM and RIFT processes. A pioneering stride in this investigative trajectory was undertaken by Gascons et al. [6], who delved the non-linear viscosity development inherent in a Liquid Composite Moulding (LCM) process. Noteworthy is their utilisation of a simplified form of the dual Arrhenius model.

Subsequently, Yang et al. and Zhou et al. [17,18] demonstrated that a more precise depiction of epoxy viscosity development can be achieved through the application of the dual split viscosity model, which delineates the evolution in terms of both physical and chemical viscosities. In a more recent study, the authors presented a numerical model for the modified viscosity incorporating variations in the viscosity of resin with time and temperature [19]. Zhang et al. [20] also used the similar viscosity development model to synthesise a high heat resistant resin.

In the current investigation, we present a meticulous infusion model that employs the non-linear dual split viscosity model for enhanced accuracy. Furthermore, the infusion process is subjected to simulation across varying temperatures to systematically analyse the influence of infusion temperature on the mould filling dynamics. One recent study [21] investigated mould filling behaviour across various initial temperatures, providing valuable insights into resin progression, which is crucial for optimising infusion processes. The current study aligns with its findings, as both explore resin movement during mould filling. However, the current study extends this work by focusing on large-scale moulds, ensuring efficient infusion for complex composite structures. It is a long-standing practice within the wind turbine manufacturing industry to initiate the infusion process at elevated temperatures due to the initially higher viscosity of epoxy resins. However, the present study demonstrates the necessity of determining the optimal temperature for mould filling to ensure efficient filling. While previous studies have explored two-step variations in viscosity and developed innovative theoretical and numerical models [17,18,19], their focus has largely remained on conceptual frameworks or simulations. Our research represents a significant advancement by experimentally demonstrating the impact of viscosity changes over time on the flow front of epoxy resins, using models established in earlier works. This experimental validation marks a novel contribution, bridging the gap between theoretical modelling and practical applications.

The findings of this study bear significant implications for both society and academia. On the societal front, the meticulous infusion model incorporating the non-linear dual split viscosity model holds promise for advancing manufacturing processes, potentially leading to more precise and efficient production methods. This has the potential to impact various industries, contributing to the optimisation of manufacturing processes and, consequently, fostering economic growth. In academia, the study focus on simulation across varying temperatures for analysing the influence of infusion temperature on mould filling dynamics contributes to the body of knowledge in the field of materials science and engineering. This research not only enhances our understanding of intricate manufacturing processes but also provides a valuable reference for future studies aiming to improve the precision and efficiency of industrial practices.

In the wind turbine industry, understanding the manufacturing processes of FRP composites is vital, especially due to the huge and complex moulds of wind turbine blades. The current study, by investigating the variations in viscosity during mould filling, provides valuable insights into optimising infusion processes for large-scale and complex moulds. By addressing factors such as temperature control and resin viscosity, the study aims to enhance process efficiency, reduce material waste, and ensure uniform resin distribution. Ultimately, the findings are expected to support the development of more durable, cost-effective wind turbine blades, enhancing sustainable energy solutions in the industry.

## 2. Material Procedure and Equipment

The epoxy resin used in the present study is EPIKOTE^TM^ MGS RIMR 035c with curing agent MGS RIMH 038, with the mix ratio of 100:28±2 by weight. The resin was originally supplied by HEXION^TM^ Epoxy, Esslingen am Neckar, GmbH, which is now Westlake Epoxy, Esslingen am Neckar, GmbH due to a change in company ownership. While the company remains the same, its name has been updated. However, our resin supply was provided under the name HEXION^TM^ Epoxy. This epoxy cures at room temperature. This is a so-called low-temperature curing epoxy, as shown in Figure 2, because the temperature remains at significantly lower levels compared to other recipes. The reinforcement mats used for this study are plain woven glass fibre mats with a weight of 290 $g/m^{2}$.

The viscosity measurements were performed in an Anton Paar MCR 301 in a cone plate configuration with a CP 50-1, which indicates a diameter of 50 mm and a 1 degree slope of the cone [22]. The shear rate was 175 per second and, for optimized temperature control, an active thermo covering was used.

Regarding the measurement of pot life, the arrangement can be characterized as semi-adiabatic: a quantity of 100 g of reactive resin-hardener mixture was placed in a paper cup. This paper cup was positioned within a fixture, closely enveloped by an insulating foam layer measuring three to four centimetres in thickness. The PT 100 temperature probe was also secured in a fixture to ensure (i) its reproducibility and (ii) its centered positioning in both radial and height coordinates.

To achieve consistent initial temperatures for comparison, the components were preheated to 29 °C overnight and subsequently mixed in a SPEEDMIXER^TM^. The duration from adding the hardener to the resin until placing the cup in the setup and initiating the measurement was maintained consistently, with a deviation of less than 1 min across all measurements. Additionally, the mixing procedure was uniform for all materials.

Once the cup containing the reactive mixture was positioned within its fixture, a 1.5 cm thick insulating foam lid was placed on top, and the measurement was commenced by inserting the sensor into the fixture.

## 3. Theory

The viscosity of epoxy resins undergoes discernible changes in response to temperature variations, driven by two distinct phenomena. Firstly, as temperature rises, molecular agitation intensifies, leading to a reduction in viscosity. Secondly, an escalation in the curing rate ensues, resulting in the formation of gigantic three-dimensional networks comprising cross-linked macromolecules.

The rheological characteristics of a typical epoxy resin can be delineated across three distinct stages, as mentioned in reference [17]. Initially, a rise in temperature induces a concurrent reduction in epoxy viscosity, primarily attributable to heightened molecular agitation. Subsequently, in the second stage, the opposing influences of the two phenomena offset each other, resulting in a relatively stable viscosity of the resin. Conversely, the third stage is characterized by a rapid surge in the reaction rate, leading to the swift proliferation of huge three-dimensional molecular structures. In this phase, the resin’s viscosity experiences a steep increase, impeding resin flow and ultimately arresting the infusion process.

For any manufacturing process involving resin transfer, the viscosity–time behaviour significantly influences the transfer time. Consequently, it is crucial to comprehend the temporal evolution of viscosity under isothermal conditions as an initial step. Common methods employed to investigate the rheological behaviour of epoxy resins include Differential Scanning Calorimetry (DSC) or Dynamic Mechanical Analysis (DMA).

**Figure 3:**
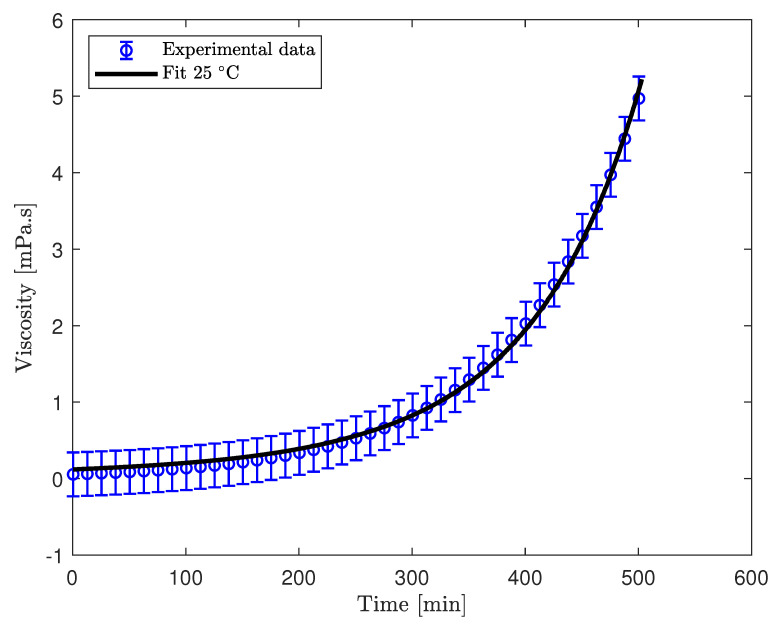
Plot of viscosity development with curing for the epoxy resin with fitted data for single exponent model presented in Equation (1).

To incorporate viscosity variations of a resin into numerical modelling of an infusion process, it is imperative to first establish a rheological model that accurately characterizes resin behaviour. Various models have been proposed in the literature to describe the rheological behaviour of epoxy resins. The majority of these expressions are formulated based on the exponential law [13,23,24]. This choice is justified by the prevalent observation that epoxy resins generally exhibit an exponential growth trend in viscosity, as depicted in Figure 3. Some researchers have opted for the modified Arrhenius equation to describe the viscosity development of epoxy resin [25,26], as indicated in Equation (1)

(1) $$\frac{\mu (t)}{\mu_{o}}={\mathrm{ae}}^{\mathrm{bt}},$$

where μ represents the viscosity of the resin at time *t*, and $\mu_{o}$ denotes the initial viscosity of the resin at t=0, with *a* and *b* being empirical parameters, the viscosity development of EPIKOTE^TM^ MGS RIMR 035c with curing agent MGS RIMH 038 at 25 °C is graphically illustrated in Figure 3 by a black solid curve. The viscosity measurements were conducted under isothermal conditions, and the data were supplied by the manufacturer. The non-linear least squares regression of Equation (1) to the measured data using MATLAB R2022b is also plotted in Figure 3 and shown with blue circles.

Notably, it is observed that the fitted curve deviates from the actual data in the initial region. Since a significant portion of Resin Transfer Moulding (RTM) mould fillings is accomplished within this time frame, this region plays a crucial role in influencing the resin infusion characteristics. Additionally, it should be emphasized that the presented data pertain to the viscosity development of the neat epoxy. In practical infusion processes, such deviations may become more pronounced, as indicated by Gascons et al. [6]. To address this, a modified model is proposed for a more accurate representation of resin viscosity development, as outlined in reference [17].

**Figure 4:**
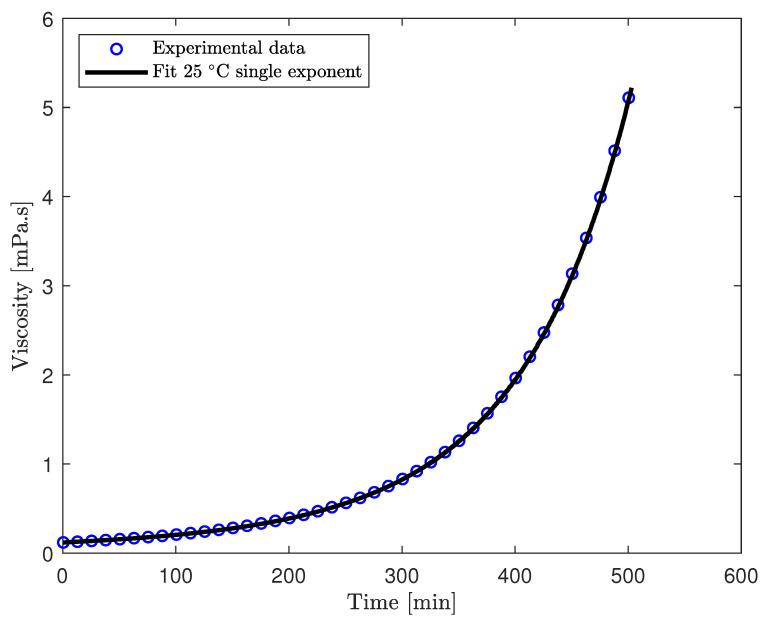
Plot of viscosity development with curing for the epoxy resin with fitted data for dual exponent model shown in Equation (3).

It is mentioned in Section 1 that the rheological behaviour of epoxy can be described by the coupling of temperature and time or degree of curing through two mechanisms. On the basis of this hypothesis, the viscosity of a typical epoxy can be split into the physical and chemical components of viscosity. The viscosity of the epoxy can then be expressed as shown in Equation (2).

(2) $$\mu =\mu_{p}+\mu_{c}$$

where $\mu_{p}$ and $\mu_{c}$ are the physical and chemical viscosities, respectively. It is assumed here that both physical and chemical viscosities follow the modified Arrhenius expression. Therefore, they can be expressed as $\mu_{p}=\mu_{o}{\mathrm{ae}}^{\mathrm{bt}}$ and $\mu_{c}=\mu_{o}{\mathrm{ce}}^{\mathrm{dt}}$. Inserting the expressions for $\mu_{p}$ and $\mu_{c}$ into Equation (2) and simplifying it, we obtain the expression shown in Equation (3).

(3) $$\frac{\mu }{\mu_{o}}={\mathrm{ae}}^{\mathrm{bt}}+{\mathrm{ce}}^{\mathrm{dt}},$$

where *a* and *c* are dimensionless empirical parameters and are represented by the ratio of physical to initial viscosity and chemical viscosity to initial viscosity, respectively. It can easily be shown that c=1−a, 0<a<1, and 0<c<1. Both *b* and *d* are empirical parameters and d>0 and b>0 for the isothermal case where b<0 for non-isothermal curing. For further details, the interested reader is referred to reference [17]. The non-linear least square regression is plotted in Figure 4 using Equation (3), which resulted in the parameters as: $a=1.351e^{-1}$, $b=1.853e^{-4}\;s^{-1}$ and $d=7.107e^{-5}\;s^{-1}$. It can be seen that the expression illustrated in Equation (3) provides an excellent fit for the development of viscosity in epoxy resin.

**Figure 5:**
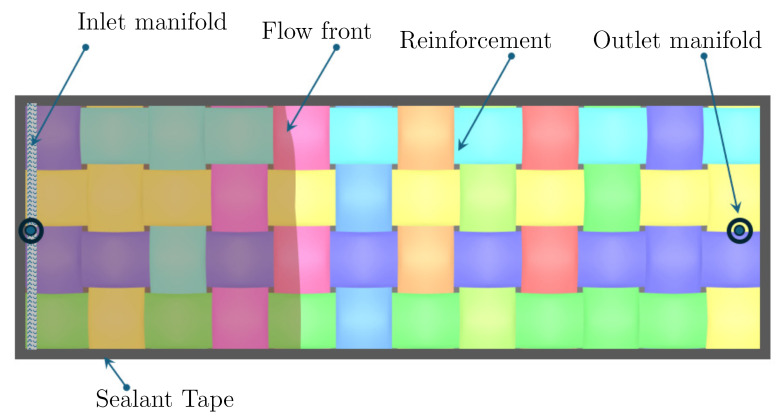
Schematic diagram of a typical infusion process. Already resin filled region is highlighted with different colour.

In this study, an analytical model is proposed by assuming a unidirectional flow through the mould in realistic conditions. The uni-directional flow assumption is justified here because the inlet gate was designed in a way that the flow of resin enters the mould parallel to the width of the mould, ensuring an in-plane flow, as shown in Figure 5. Flow through the thickness of the mats is neglected in this model, which is a valid assumption because the thickness of the mould is at least two orders of magnitude less than the planar dimensions of the layup of reinforcement mats. The conditions mentioned above are realistic and are valid for a wide range of practical cases in the infusion process. The constant inlet pressure boundary condition is also ensured during the process.

Darcy’s law is used to express the flow through a porous medium. The one-dimensional form of Darcy’s law is depicted in Equation (4)

(4) $$v\left(t\right)=-\frac{\kappa }{\mu }\frac{\mathrm{dp}}{\mathrm{dx}}=\frac{\kappa }{\mu }\frac{\Delta P}{x(t)-x(0)},$$

where v(t) is the volume-averaged velocity of the penetrating liquid in the direction of the flow front travel, κ is the permeability, ΔP is the pressure gradient, and μ is the dynamic viscosity of the liquid, as mentioned in Equation (3).

The Darcy velocity is defined as the average velocity of the fluid through the volume element of a porous medium consisting of both fluid and solid volumes. The Darcy velocity can be related to the average intrinsic velocity V=dx/dt of the fluid, as shown in Equation (5), through the porosity of the medium [27]. Where the intrinsic velocity is the average velocity of the fluid over the fluid part of the porous medium.

(5) $$v\left(t\right)=\phi \frac{\mathrm{dx}}{\mathrm{dt}},$$

where ϕ is the porosity of the medium which can be expressed in terms of the fibre volume fraction as $1-V_{f}$.

Inserting the expressions from Equations (3) and (5) into Equation (4) and integrating with respect to time *t*, one obtains the expression mentioned in Equation (6)

(6) $$x\left(t\right)={\left(\frac{2\kappa \Delta P}{\left(1-V_{f}\right)\mu_{o}}\int_{0}^{t}\frac{\mathrm{dt}}{{\mathrm{ae}}^{\mathrm{bt}}+{\mathrm{ce}}^{\mathrm{dt}}}\right)}^{1/2}$$

With the knowledge of empirical parameters, such as the initial dynamic viscosity of the fluid, permeability of the reinforcement mats, the fibre volume fraction, the differential pressure, and the specific resin parameters a,b,c, and *d*, one can use Equation (6) to compute the flow front position at a specific infusion time *t*.

## 4. Experimental

Figure 5 depicts the image of the infusion experiment, illustrating the flow front position. For the experimental setup, an aluminium plate is used as the rigid base to support the reinforcement mats. Among the other possibilities, it is common practice in the wind turbine industry to use Teflon sheets on the surface of the mould as a release agent. Therefore, A Teflon sheet containing adhesive on one side was placed on the surface of the Aluminium plate to act as a release agent. Four layers of 250 mm × 1000 mm were cut from the plain woven glass fibre mats. The sheets were carefully laid up on top of each other to avoid any wrinkles. Sealant tape was applied to the periphery of the reinforcement mats.

Subsequently, inlet and outlet manifolds were positioned at the remote ends of the mould. A spiral-cut tube was employed at the inlet manifold to induce a planar flow. A vacuum bag was then meticulously placed over the mould, pressing it onto the previously applied sealant tape to establish a sealed cavity for vacuum retention. To closely observe the real effects of resin flow, no auxiliary elements such as channels, runners, etc., were utilized to aid resin flow. This configuration emulates the infusion process of a large mould within the confines of a small laboratory setup.

Careful attention was given to the application of the vacuum bag to prevent any wrinkles, thereby enhancing the visibility of the flow front. Although this setup may impose strain on the vacuum bag, measures were taken to ensure that it did not rupture due to such stresses. To facilitate resin flow, 8 mm diameter silicon tubes were inserted into the inlet and outlet manifolds and sealed with the aid of sealant tape. A rotary vacuum pump was employed for vacuum application. Both the hardener and resin were maintained at 25 °C prior to mixing to achieve uniform initial infusion temperature for the resin. The resin and hardener were measured using a weighing scale, and mixing was achieved manually.

As soon as the ingredients were mixed thoroughly, the resin was degassed using the vacuum and the infusion started without any delay. The pot containing the epoxy mixture was placed in a water container to maintain the temperature at 25 ± 1 °C. Throughout the infusion process, the temperature of the epoxy mixture was closely monitored using the digital temperature logger, Picolog. After applying the vacuum, the thickness of the layup was measured at various locations. The same procedure was repeated after the infusion process. The data could then be used to compute the $V_{f}$ of the laminate [6]. The average value of $V_{f}$ was found to be 58%. Throughout the infusion process, the surface temperature of the mould was also monitored. A camera was fitted on an overhanging stand on top of the mould to record the infusion process. During the infusion process, the flow front position was recorded, and the corresponding time was also noted during the whole infusion process.

The reproducibility of results in the resin infusion process can be influenced by several experimental factors. Some important factors are listed here:

- Vacuum Level: Variations in the vacuum pressure during the infusion process can significantly affect resin flow dynamics, particularly in terms of filling time and uniformity. To ensure reproducibility, we maintained a consistent vacuum level of 0.2 bar throughout all experiments, verified using the calibrated pressure gauge.
- Reinforcement Configuration: The placement, orientation, and type of reinforcement material (e.g., fibre volume fraction, permeability) impact resin flow paths and infusion efficiency. In our study, we standardized the reinforcement configuration by using and ensuring consistent layup across all experiments.
- Resin Temperature: As temperature affects viscosity, the resin was preconditioned to a controlled temperature of 25 °C prior to infusion to minimize variability.
- Mould Design and Seal Integrity: The design of the mould and the quality of its seals were carefully controlled to avoid leakage, which could affect both flow behaviour and repeatability.

However, as concluded in a benchmark exercise by Arbter et al., the variation in experimental results is mainly due to human factors, highlighting the importance of skilled personnel for consistent data in permeability testing [28].

## 5. Results and Discussion

The dual-exponent viscosity model, articulated in Equation (3), exhibits exemplary conformity with the empirical curing data, as depicted in Figure 4. Consequently, for validation purposes, the dual-exponent viscosity model was integrated into the one-dimensional formulation of Darcy’s law. The application of this methodology can be readily extended to encompass more intricate scenarios for enhanced realism.

**Figure 6:**
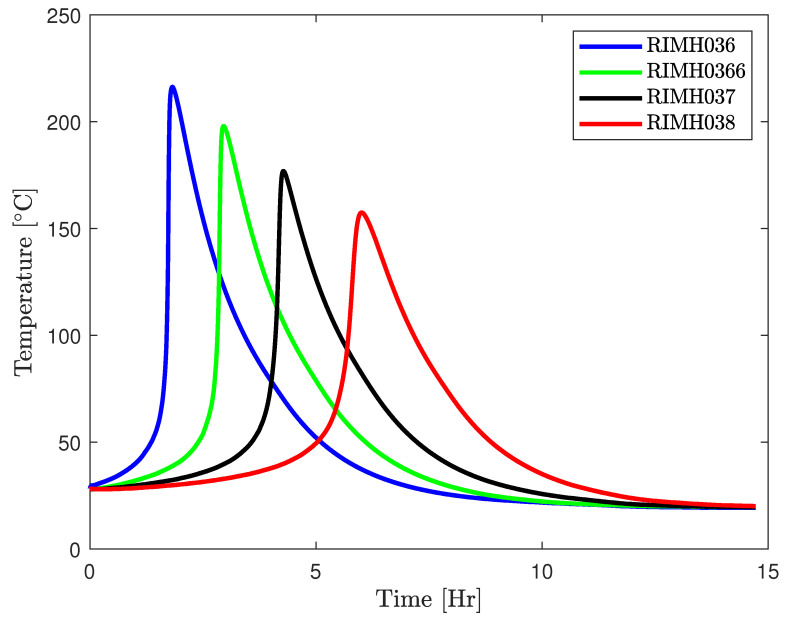
Plot of temperature development of RIMR 035c epoxy resin with four different hardeners under insulated setup [29].

In the present investigation, a low-heat-generating recipe is employed, and the utilization of a water bath for the resin-hardener reservoir helps mitigate temperature fluctuations. Additionally, due to the sufficiently large surface area of the mould, the dissipation of heat is substantial, resulting in negligible temperature variations. Under these circumstances, it is justifiable to consider the infusion process as isothermal. This assertion is supported by the findings illustrated in Figure 6, where identical epoxy formulations are examined for both pot life and temperature evolution within an insulated configuration. It is apparent that, in such conditions, the epoxy can attain higher temperatures due to the absence of heat dissipation. This methodology can be extrapolated to other epoxy formulations with high heat generation, provided an efficient heat exchange system is implemented in the mould.

MATLAB R2022b was utilized for simulating the infusion process based on the equation referenced in Equation (6). The empirical parameters, denoted as *a*, *b*, and *d*, were determined through non-linear least squares curve fitting, as elaborated in Section 3. The disparity in pressure, denoted as ΔP, is documented under the process conditions, wherein $V_{f}$ can be derived from the variation in thickness of the layup before and after infusion, along with the mould dimensions. Another essential parameter is the permeability, denoted as κ. The permeability can be computed by assuming the epoxy viscosity to remain constant over a small time increment.

**Figure 7:**
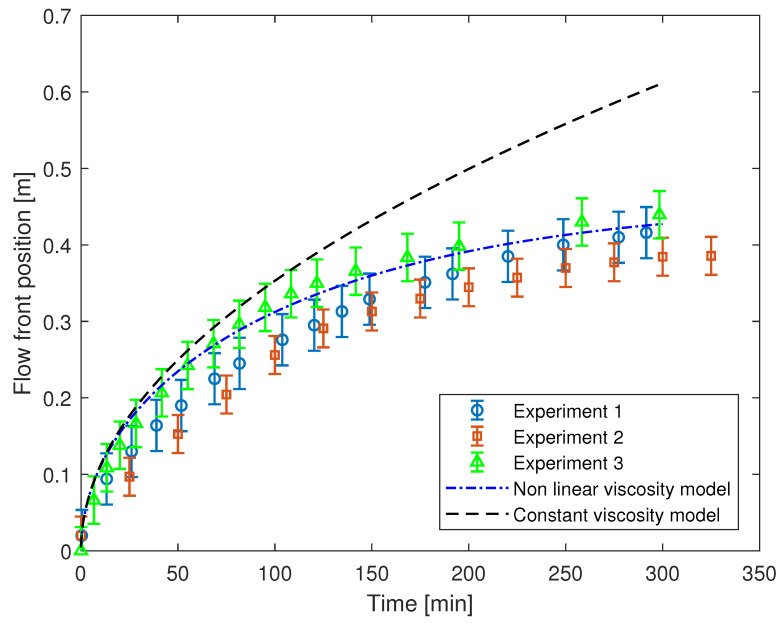
Comparison of the flow front position for real infusion of epoxy and the model presented in Equation (6).

For this objective, the selected time window corresponds to the initial phase of the infusion process, during which the cross-linking process progresses at a notably slow pace. Employing a constant viscosity under these conditions would be a justified assumption [28]. Consequently, an ideal unidirectional (UD) infusion model can be represented, thereby applying the general form of Darcy’s law for UD flow, as specified in Equation (7).

(7) $$\kappa =\frac{v\mu L}{\Delta P}$$

where *v* is the volume-averaged velocity, *L* is the distance travelled by the impregnating resin at a given time, and initial viscosity is employed as the constant viscosity. Therefore, using Equation (7), the permeability was found to be $5.3782\;\cdot \;10^{-12}\;m^{2}$. The value of the initial viscosity of the epoxy is taken from the experimentally measured values. The flow front position was simulated in MATLAB using Equation (6) for each time step. The integral in Equation (6) is solved numerically and applied for each time step. The flow front position is plotted for each time step in Figure 7. The flow front position recorded during the experiment with recorded time steps is also plotted in the graph with small circles. Overall, an excellent agreement is found between the simulation and the real infusion data. It is worth noting that the overall mould filling seems to be a bit slower. However, it may be noted that in an attempt to mimic the infusion for the huge parts, no aid was used in the present study to facilitate the flow of resin within the mould, including runner channels and infusion mesh. Mould filling data for the constant viscosity are also plotted in Figure 7. Clearly, the constant viscosity assumption would produce a significant error in the simulation of the infusion process, especially if the mould filling takes longer than an hour.

Another pivotal factor demanding careful consideration in facilitating an efficient infusion process pertains to the optimal temperature at the commencement of said infusion process. In contemporary industrial practice, it has become customary to initiate the infusion at an elevated temperature, given that, initially, the viscosity of the resin is lower under comparatively higher thermal conditions. Nevertheless, this initial advantage is soon offset as the heightened temperature accelerates the cross-linking process at a greater rate. This phenomenon arises due to the exothermic nature of the process, wherein the enhancement of cross-linking occurs rapidly, resulting in a more pronounced increase in resin viscosity. Consequently, the temperature at which the infusion commences assumes a critical role in achieving a successful yet efficient infusion process.

**Figure 8:**
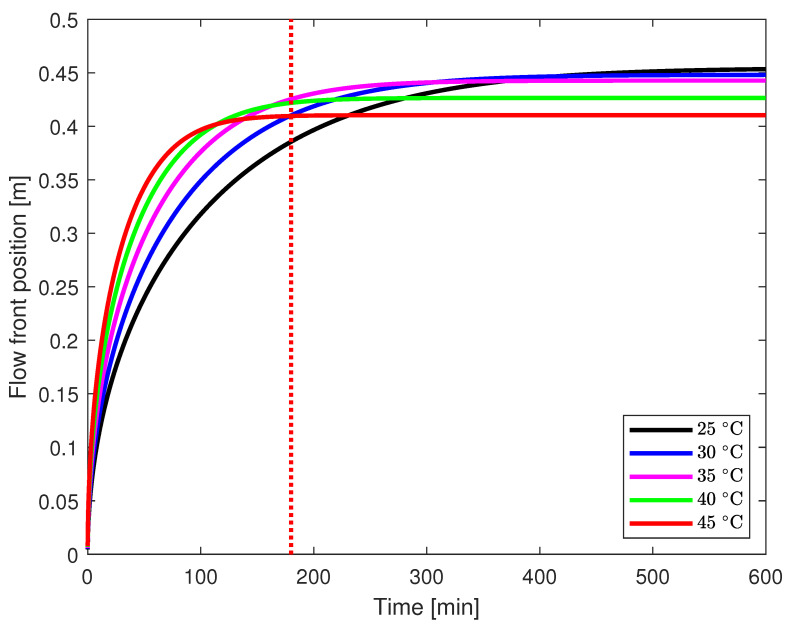
Plot of the flow front with time for UD flow case. The dotted red line exemplifies a three-hour mould filling time-frame. Infusion at 40 or 45 °C results in an early gelation point causing an incomplete mould filling, while 25 °C causes inefficient filling and extended duration. An optimal starting temperature of 35 °C ensures efficient mould filling.

In the current investigation, simulations were conducted to assess the flow front position at various temperatures ranging from 25 °C to 45 °C. The outcomes of these simulations are graphically depicted in Figure 8. Notably, despite the resin’s higher viscosity at the outset at 25 °C, the cross-linking rate is comparatively sluggish, leading to a more gradual increase in viscosity. Conversely, commencing the infusion at a higher temperature, such as 45 °C, results in rapid initial mould filling. However, this also leads to an early onset of the gelation point, prematurely halting resin movement. A recent study [21] provides valuable insights into mould filling behaviour across an initial mould filling temperature range of 25–125 °C, with a maximum filling time of 300 s. This work aligns well with our study, as both emphasize the importance of resin flowability before reaching the gelation point, particularly for complex moulds. Our study extends this understanding by demonstrating similar resin behaviour at lower temperatures, highlighting the need for tailored resin formulations based on mould size and infusion time. By focusing on large-scale components like wind turbine blades, our work complements and builds upon their findings in practical industrial applications.

To elucidate further, let us consider a scenario involving a relatively large mould that requires approximately three hours to fill. A dotted red vertical line representing this timeframe is delineated in Figure 8. It is evident that if the infusion commences at 40 or 45 °C, the resin will reach its pot life well before the two-hour mark, resulting in incomplete mould filling. Conversely, initiating the infusion at 25 °C leads to inefficient filling and a prolonged duration for completion. Optimal efficiency in the infusion process for this specific epoxy recipe and mould size may be achieved by commencing the infusion at 35 °C.

It is crucial to account for both the physical and chemical phenomena when determining the infusion start temperature. Physically, higher temperatures reduce the resin’s viscosity, promoting smoother and faster resin flow during the initial stages of the infusion. However, chemically, the exothermic nature of the curing reaction means that elevated temperatures also accelerate the cross-linking process, which increases viscosity over time and may prematurely hinder resin flow. Therefore, balancing these factors is essential to avoid early gelation while ensuring efficient mould filling, ultimately enabling a successful and controlled infusion process.

Building upon this discussion, an effective mould filling strategy is proposed. The infusion process can be enhanced by initiating it at a higher temperature, such as 45 °C. Subsequently, after a predetermined duration, temperature reduction can be implemented using a heat exchanger to maintain lower temperature levels. This approach serves to decelerate the cross-linking process, thereby extending the time-frame available for mould filling.

## 6. Conclusions

In the presented work, an accurate model is presented to incorporate the non-linear development of epoxy resin in the infusion simulations. A dual-exponent epoxy viscosity model separating the physical and chemical phenomenon of viscosity development is employed in the UD Darcy’s law. The model is compared with the experimental infusion study. An excellent agreement is found between the non-linear viscosity model and the experimental study after the boundary conditions have reached a constant state. It is shown that assuming a constant viscosity for comparatively larger moulds may lead to significant errors.

A comparative study of the effect of temperature at the start of the infusion is presented in the current study. It is shown that starting the infusion process at a higher temperature, when the viscosity of the epoxy resin is lower, may lead to erroneous results. It is vital to take both the physical and chemical phenomena into consideration before deciding the infusion start temperature. It is also proposed that the infusion process can be completed in steps by starting at the higher temperature and, after a calculated time, decreasing the mould temperature in a systematic way to achieve the most optimum mould filling.

## Data Availability

The original contributions presented in this study are included in the article. Further inquiries can be directed to the corresponding authors.
